# Midwives’ Perceptions of Promoting Pregnancy Vaccines in Wales: Identifying Factors Influencing Vaccine Uptake Using the COM‐B Framework

**DOI:** 10.1002/puh2.70114

**Published:** 2025-09-07

**Authors:** Kate Lloyd, Sara Jones

**Affiliations:** ^1^ Swansea University Swansea UK

**Keywords:** capability, opportunity, motivation‐behaviour (COM‐B), midwives, perceptions, pregnancy, vaccination, Wales

## Abstract

**Introduction:**

Vaccinations are vital for global health; however, since the onset of the COVID‐19 pandemic in 2020, there has been a notable decline in maternal vaccine acceptance in Wales, UK. It is a key part of a midwife's role to promote vaccine uptake in pregnancy. Therefore, gaining an understanding of midwives’ perceptions of the issue is crucial for identifying factors influencing vaccine uptake in Wales.

**Methods:**

A quantitative, cross‐sectional descriptive survey was conducted amongst 143 registered midwives working in Wales using the Capability, Opportunity, Motivation‐Behaviour (COM‐B) approach to identify factors affecting vaccine uptake.

**Results:**

Midwives lacked training and confidence to promote vaccines, and some noted language barriers as a problem to providing equitable care. Other problems included lack of access to vaccination appointments and inconsistencies in vaccine promotion depending on care provider. Midwives perceived vaccine hesitancy to be high, with 71% saying they thought vaccine hesitancy was common amongst pregnant women and 40% saying they thought it was common amongst their colleagues.

**Conclusion:**

A national approach is required to ensure effective vaccination training amongst midwives in Wales, as well as improved availability of language diverse resources. Additional qualitative research is needed in Wales and the United Kingdom to further understand vaccine hesitancy and barriers to promoting vaccine uptake in pregnancy.

## Graphical abstract

GRAPHICAL ABSTRACT

This study reports on midwives working in Wales views regarding pregnancy vaccination. Midwives in Wales face challenges promoting vaccination, including limited training, language barriers, time constraints and inconsistent and inaccessible care. Many perceived there to be high vaccine hesitancy among pregnant women and, to a lesser degree, among colleagues.

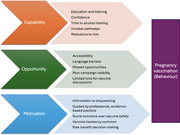

## 1 Background

Vaccines are recognised as the second most effective public health intervention after clean water, and in pregnancy they provide protection for the mother and the unborn baby through passive immunity [1]. In Wales, UK, the National Health Service (NHS) delivers the pregnancy vaccination schedule, which includes vaccines against influenza, pertussis, and respiratory syncytial virus (RSV), however at the time of this study the schedule also included the COVID‐19 vaccine, [2]. This schedule is recommended by the Joint Committee on Vaccination and Immunisation (JCVI), a scientific advisory body which advises the United Kingdom government and devolved nations, including Wales [3].

Pregnant women are at an increased risk of death from influenza. Between 2009 and 2012 there were 29 deaths of pregnant women and women up to 6 weeks postpartum associated with influenza in the United Kingdom [4]. More than half of these deaths occurred after a vaccine had become available in 2010. Further reports have reported an additional five maternal deaths, all of which were of unvaccinated women [5, 6, 7]. The maternal pertussis vaccination programme was introduced in 2012 in response to an outbreak with severe outcomes in infants under 6 months [8]. Between 2002 and 2011, 48 pertussis‐related infant deaths occurred, 41 in babies too young to be vaccinated [9]. Since the programme's introduction, 31 infant deaths have been reported, 25 in babies whose mothers were unvaccinated during pregnancy [10]. The COVID‐19 vaccine was introduced to pregnant women in 2021 during a pandemic when pregnant women were added to the clinical risk group; however, a recent JCVI statement advises a significant reduction to the COVID‐19 programme for 2025, which does not recommend routine COVID‐19 vaccination for pregnant women in the United Kingdom due to significantly decreased risks associated with the virus in the last 18 months [11, 12]. From 1 September 2024, the RSV vaccine has been introduced to the United Kingdom schedule. RSV contributes to several hospitalisations in infants under 1 and can cause serious respiratory infection [13]. The Vaccine Development and Evaluation Centre (VDEC) has also recently announced the development of a new maternal Group B Streptococcus vaccine [14]. The measles, mumps and rubella vaccine (MMR) is not given in pregnancy but are recommended for women planning to conceive if not received in childhood. Rubella and measles infections in pregnancy may cause miscarriage, stillbirth, pre‐term birth and congenital abnormalities [15, 16].

Despite the recognised benefits of maternal vaccination, uptake in Wales has declined. A Point of Delivery Survey reports on a snapshot of vaccination coverage across Wales taken from women delivering babies in each health board over a 5‐day period in January each year. The report highlighted that uptake of the influenza vaccine in pregnancy was only 60.9% in 2023/24 compared to 78.5% in 2019/20 [17]. In 2023/24, uptake of the pertussis vaccine in pregnancy was 70% compared to 80% the previous year [18]. Further data suggests in 2021–2022, uptake of the COVID vaccine during pregnancy in Wales was only around 1 in 3 [19]. In 2022 the Welsh National Immunisation Framework was published, which aimed to improve uptake of vaccines in Wales and to deliver ‘world leading outcomes in vaccine preventable disease’ with ‘high uptake’ of vaccines, though specific targets for uptake are not outlined [20].

Vaccine refusal can be influenced by concerns about long‐term effects on the baby and a preference to wait until after pregnancy, with factors such as anxiety, inconsistent information, misinformation and the influence of social media and alternative parenting contributing to vaccination hesitancy [19, 21, 22]. Anxiety around vaccines in pregnancy may be very high, with one respondent in a UK and Irish qualitative study calling deciding whether to have a vaccine *‘the hardest decision of my life’* [23]. Recent linked‐data research of over 795,000 babies in the United Kingdom has shown that women who accept pregnancy vaccinations are also 40% more likely to vaccinate their child [24]. Therefore, understanding pregnant women's decisions around vaccinations is complex, important and may have long‐term consequences. Vaccine hesitancy is a serious threat to vaccination programmes and has been identified as one of the WHO's [25] 10 global health threats.

In the United Kingdom almost all pregnant women will see a midwife, and some may only see a midwife as their healthcare professional throughout their pregnancy and birth. Promoting vaccination in pregnancy is part of a midwife's role, and midwives significantly influence vaccination decision making [26, 27, 28, 29]. Prior research with midwives in the United Kingdom and other high‐income countries has revealed several factors influencing midwives’ effectiveness in promoting maternal vaccine uptake. Lack of adequate training and experience negatively affects midwives’ confidence in promoting vaccines [30, 31, 32], and pregnant women have also reported lack of information from midwives about vaccines leading to uncertainty [33]. There may be operational and workforce barriers to vaccine uptake [34]. Supportive policies and guidance have been identified as essential in facilitating maternal vaccination programmes [35, 36], though these need to be clearly communicated to front‐line staff. For example, in an Australian study, participants described a delay during the COVID‐19 pandemic in aligning clinical practice with guidance, with over half of healthcare professionals surveyed reporting being unsure about recommending the COVID‐19 vaccination to breastfeeding women, despite national guidance being available at the time [36].

Individual midwives’ personal attitudes towards vaccination are also important to understand. In a qualitative study in Spain, Arreciado Marañón [37] found that midwives were frequently asked what they would do personally, with some participants sharing their own vaccination status with patients. In the United Kingdom, Vishram et al. [31] found that health visitors, practice nurses and midwives who personally accepted or hypothetically would accept a vaccine if eligible were more likely to recommend it. They also found that of these three professions, midwives were least likely to accept a vaccine. Cultural disparities have also been observed. In Odejinmi et al.’s [38] study in London, midwives of Black ethnicities were four times less likely to accept the COVID‐19 vaccination compared to those of White ethnicities, and they were also less likely to accept the influenza vaccine. Concerns surrounded porcine gelatine content and perceived increased risk of adverse effects of the vaccines on ethnic minority women. Historical medical mistrust has also been documented as influential on vaccine hesitancy amongst the Black British population [34].

To date no research has explored midwives’ attitudes and perceptions of pregnancy vaccination in Wales. This research aimed to address this gap. Our research questions were:

1. What are the practices and attitudes of midwives in Wales towards pregnancy vaccination?
2. What are the perceived factors influencing pregnancy vaccination in Wales?

## 2 Methods

### 2.1 Design, Participants and Materials

**FIGURE 1:**
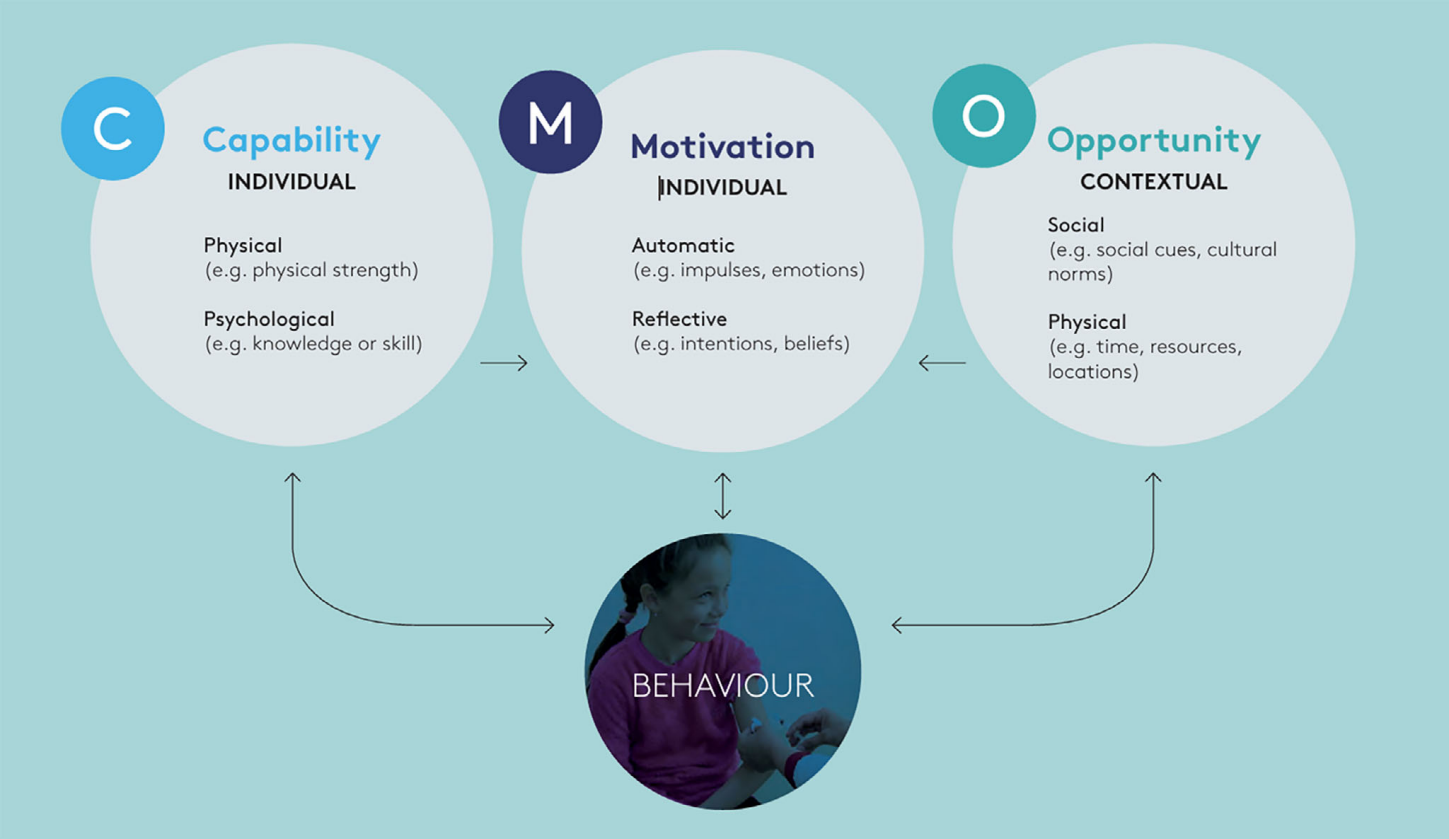
The COM‐B framework. *Source:* This figure was extracted from the World Health Organization [39]. This work is available under the Creative Commons Attribution NonCommercial‐ShareAlike 3.0 IGO licence (CC BY‐NC‐SA 3.0 IGO; https://creativecommons.org/licenses/by‐ncsa/3.0/igo). Under the terms of this licence, material may be copied, redistributed and adapted for non‐commercial purposes, provided the work is appropriately cited, as indicated below. In any use of this work, there should be no suggestion that WHO endorses any specific organization, products or services.

The research used a simple cross‐sectional survey design. All midwives working in Wales were eligible to participate in the study via an online questionnaire via the Qualtrics platform. The survey contained open and closed questions, including several 5‐point Likert scale style questions (strongly agree/somewhat agree/neither agree nor disagree/somewhat disagree/strongly disagree), assessing agreement with statements relating to midwives’ vaccine practice and attitudes, framed around the capability, opportunity, motivation‐behaviour (COM‐B) model (see Figure 1). COM‐B identifies the capability, opportunity and motivational (COM) factors required for a behaviour (B) (in this case vaccination) to happen and is commonly used for designing and evaluating health behaviour change strategies [39]. The questionnaire included questions which explored the following:

1. Demographic details (geographical location of work, length of service, type of work, e.g., antenatal/postnatal/labour).
2. Vaccination training history.
3. Midwives’ perceived confidence and capability to support women with pregnancy vaccination decisions.
4. Midwives’ perceived operational barriers to pregnancy vaccination.
5. Resources used to support pregnancy vaccination.
6. Midwives’ own attitudes toward vaccinations in pregnancy and perceptions of risk for vaccinated diseases (COVID‐19, influenza, pertussis, measles, mumps, rubella and RSV) for both mother and baby independently.
7. Midwives’ perceptions of pregnant women's, their partners’, their families’ and their colleagues’ beliefs about vaccines and their perceived degree of vaccine hesitancy in each of these groups.

The questionnaire was pilot tested with registered nurses who work with the lead researcher.

### 2.2 Procedure

Data were collected in July and August 2024. Advertisements containing brief details of the study and eligibility criteria were placed on the researchers’ social media pages with encouragement for midwives to share the post. Anyone interested could click on a link which took them to the study information sheet and consent questions. An eligibility question (‘Are you a midwife working in Wales?’) was placed with the consent questions. Only after consent was agreed and eligibility confirmed did the full questionnaire load. Once completed, a debrief statement appeared thanking participants with information on how to contact local vaccination teams for support. Ethical approval was granted by **[redacted for peer review]** research ethics committee.

The sampling frame comprised all midwives registered with the Nursing and Midwifery Council in Wales in 2023 (*n* = 1904). Using a sample size calculator [40], a target of 320 participants was set to achieve a 95% confidence level with a 5% margin of error.

### 2.3 Analysis

Quantitative data were analysed by [author name—redacted for peer review] supervised by [author name] using SPSS version 29 to compute descriptive statistics from answers to closed questions. A simple descriptive analysis was conducted on qualitative data from the short open‐ended answers [41]. For this, the first author immersed themselves in the data and identified initial themes and subthemes which were reviewed by the second author. Where disagreement occurred, themes were discussed until agreed.

## 3 Results

A total of 154 midwives in Wales took part; however, 11 surveys were grossly incomplete and were removed from analysis, leaving 143 responses which were either complete (*n* = 108) or partially completed (*n* = 35). The total number of midwives answering each question therefore varied.

### 3.1 Demographics

Midwives from across all seven geographical regions in Wales took part. Most midwives had been qualified over 5 years (*n* = 105, 73.41%). Midwives were asked when they typically delivered maternity care (midwives could tick multiple options or write if ‘other’). The most common stages were the second trimester (*n* = 83, 58%), the third trimester (*n* = 91, 63.6%), labour and delivery (*n* = 97, 67.8%) and post‐natal care (*n* = 95, 66.4%). Other roles (textbox) included ‘non‐clinical’ roles (*n* = 4), ‘working throughout pregnancy’ (*n* = 4), ‘community’ (*n* = 3), ‘ante‐natal clinic’ (*n* = 2), ‘sonography’ (*n* = 1), ‘education’ (*n* = 1), ‘dual registration’ (*n* = 1) and a ‘specialist’ (*n* = 1).

**FIGURE 2:**
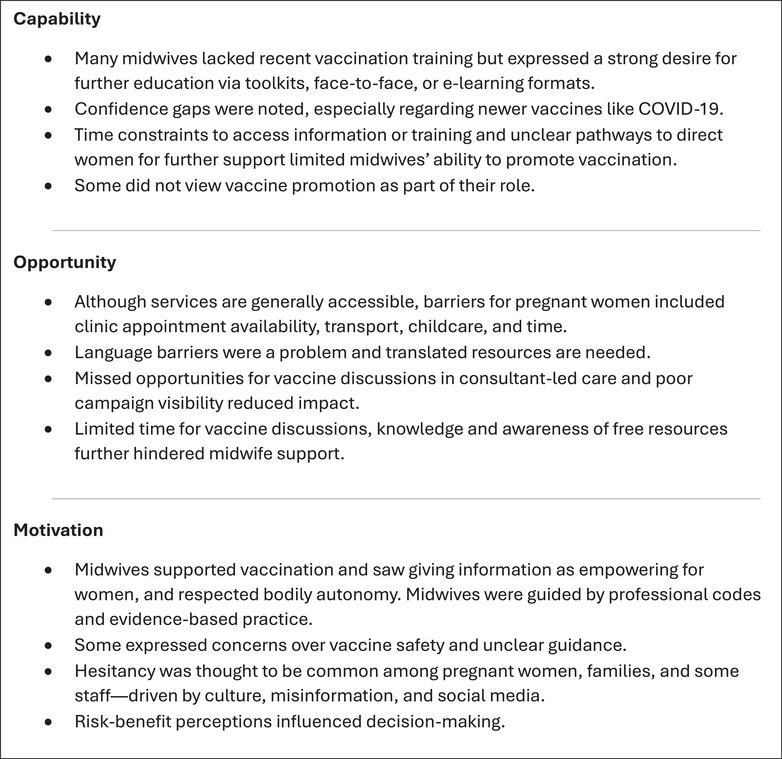
Summary of midwives’ perspectives of factors influencing pregnancy vaccine uptake in Wales.

Results were organised according to the COM‐B framework. Figure 2 shows a summary of the findings.

### 3.2 Capability

Most midwives agreed that offering maternal vaccines is part of their role (*n* = 113, 86.3%). However, many reported either not receiving any vaccination training during their pre‐registration education (*n* = 66, 46.2%) or were unsure if they had (*n* = 24, 16.8%). Less than half of midwives recalled receiving post‐registration vaccination training (*n* = 65, 45.5%). Of those who had training, around half (*n* = 35, 53.8%) were trained within the past 2 years.

Confidence in advising women on vaccines varied by vaccine type: see Table 1. Midwives felt more confident discussing the influenza (*n* = 102, 77.8%) and pertussis (*n* = 102, 77.8%) vaccines compared to the COVID‐19 vaccine (*n* = 54, 48.8%). Two thirds of midwives agreed that they were confident in their ability to gain knowledge and provide information if a new vaccine was recommended during pregnancy (*n* = 88, 67.2%). Just under two‐thirds of midwives (*n* = 78, 59.6%) felt confident in accessing resources and knowledge to address common vaccine misconceptions, but only around a quarter (*n* = 34, 26.2%) felt they had enough time to access these learning materials. The majority (*n* = 107, 81. 1%) said they wanted to receive further vaccine education. The preferred training option was a pregnancy vaccination toolkit (*n* = 79, 59.9%), followed by face‐to‐face training (*n* = 55, 41.6%) and eLearning modules (*n* = 55, 41.6%).

**TABLE 1:** Midwives’ confidence in discussing vaccines.

Statement	Agreement scale	*n*	%
I perceive offering vaccine information in pregnancy is part of my role (*N* = 131)	Strongly disagree	7	5.3
	Somewhat disagree	3	2.3
	Neither agree nor disagree	8	6.1
	Somewhat agree	25	19.1
	Strongly agree	88	67.2
I am confident in my ability to discuss the benefits and risks of the COVID‐19 vaccination in pregnancy (*N* = 131)	Strongly disagree	17	13
	Somewhat disagree	27	20.6
	Neither agree nor disagree	23	17.6
	Somewhat agree	38	29
	Strongly agree	26	19.8
I am confident in my ability to discuss the benefits and risks of the influenza vaccination in pregnancy (*N *= 131)	Strongly disagree	8	6.1
	Somewhat disagree	13	9.9
	Neither agree nor disagree	8	6.1
	Somewhat agree	62	47.3
	Strongly agree	40	30.5
I am confident in my ability to discuss the benefits and risks of the pertussis vaccination in pregnancy (*N *= 131)	Strongly disagree	7	5.3
	Somewhat disagree	12	9.2
	Neither agree nor disagree	10	7.6
	Somewhat agree	51	38.9
	Strongly agree	51	38.9
If there was a new vaccination recommended in pregnancy, I would feel confident in my capability to gain knowledge to deliver the information to my patients (*N *= 127)	Strongly disagree	9	6.9
	Somewhat disagree	13	9.9
	Neither agree nor disagree	21	16
	Somewhat agree	57	43.5
	Strongly agree	31	23.7

### 3.3 Opportunity

Around two thirds of midwives agreed that vaccination services within their region are easily accessible for pregnant women (*n* = 87, 65.9%). However, over half identified difficulty accessing clinic appointments as the main operational barrier to women accessing vaccines (*n* = 67, 51.5%), followed by transportation challenges (*n* = 53, 40.7%), childcare commitments (*n* = 47, 36.2%) and lack of time to attend appointments (*n* = 43, 33.1%). For context, it is important to note that in Wales pregnancy vaccinations are typically given at a primary care or general practice clinic by a practice nurse, without a GP appointment. Some areas have trialled midwifery led vaccination clinics. One participant identified the location of the appointment at the clinic as being a barrier; however, another said that ‘*in my health board midwives deliver the vaccines so there is no need for [women] to go to their GP* [clinic]*’*. Midwives who selected ‘other’ (*n* = 18, 13.8%) could provide further details in an open text box, with 16 doing this. The most identified barrier was language (*n* = 8), with one participant noting ‘*language barriers are a huge problem; we don't always have information in their first language’*, and another said, ‘*even with a translator I have found some women do not understand’*. Vaccine discussions were also dependent on who is delivering care. For example, one participant said, *‘it depends on the midwife informing women and if that fails sometimes, they can be missed’* and another noted* ‘consultant‐led women* [i.e., those not under full midwife‐led care] *have less appointments with community midwives so don't have much discussion around vaccines.’* Visibility of vaccine campaigns was also identified as a barrier (*n* = 2); one participant said, *‘vaccines are not well advertised’*, whereas another stated *‘promotion of maternal vaccines seems to be fragmented’*.

Midwives were asked about operational barriers which affect their ability to promote vaccines. The most common barriers were midwives’ knowledge (*n* = 46, 35.4%) and time (*n* = 36, 27.7%) to discuss vaccines in depth. Amongst those who selected ‘other’ (*n* = 20, 15.4%), free text answers revealed that midwives providing care later in pregnancy did not perceive vaccination discussions as part of their role (*n* = 9). One participant also stated that *‘midwives are generally not in a position to signpost as they do not know where to go’*. Three quarters of midwives weren't aware that vaccination resources from Public Health Wales could be ordered for free (*n* = 99, 78%).

### 3.4 Motivation

Most midwives (*n* = 104, 89.6%) agreed that supporting maternal vaccination aligns with their own commitment to public health. Likewise, almost all midwives believed in the importance of protecting both mother and baby from vaccine preventable diseases (*n* = 110, 94.9%) and agreed that this contributes to healthier outcomes for both (*n* = 106, 92.2%). Nearly all (*n* = 108, 93.9%) felt that providing information on maternal vaccination empowers women's decision making.

Most midwives (*n* = 102, 87.9%) trusted that evidence‐based research shows vaccines are safe and effective for pregnant women. However, around a quarter also (*n* = 26, 26.7%) expressed concerns about vaccine safety during pregnancy and were cautious when promoting. Over half (*n* = 67, 57.8%) of midwives agreed that a lack of clear guidance or conflicting information makes promoting vaccines challenging. A small minority of midwives (*n* =  7, 6.1%) felt vaccines conflicted with their personal or cultural beliefs.

The midwives were asked to rate the perceived seriousness of COVID‐19, influenza, pertussis, measles, mumps, RSV and rubella for the pregnant woman, the unborn foetus and the newborn baby independently using a 5‐point Likert scale (very serious to not serious at all). Please see Table 2. For pregnant women, COVID‐19 (*n* = 91, 77.5%) and influenza (*n* = 101, 87.1%) were less likely to be perceived as serious compared with measles (*n* = 107, 93.1%). Regarding the unborn foetus, COVID‐19 (*n* = 70, 60.4%) and influenza (*n* = 84, 72.2%) were again less likely to be perceived as serious, whereas rubella was viewed as the most serious (*n* = 107, 92.3%). For newborn babies, the perceived seriousness of all diseases increased. Although the perception of COVID‐19 seriousness was higher (*n* = 101, 87.1%), compared with other populations, it remained the lowest compared to other diseases. Pertussis was perceived as the most serious illness (*n* = 115, 99.1%) in newborns. Midwives were also asked to rate the perceived negative effects of maternal vaccines on pregnant women, unborn foetus and the newborn. 38.7% (*n* = 38) of midwives perceived that negative effects of vaccines on a pregnant women could be serious. Similarly, 36.3% (*n* = 36) perceived that negative effects of vaccines on an unborn foetus could be serious. For newborn babies, 40.8% (*n* = 40) perceived that negative effects of vaccines could be serious.

**TABLE 2:** Midwives’ perceived seriousness of diseases.

		For pregnant women		For an unborn foetus		For a newborn baby	
**Disease**	**Perceived seriousness**	*n*	%	*n*	%	*n*	%
COVID‐19 (*N *= 116)	Very serious	40	34.5	32	27.6	62	53.4
	Fairly serious	51	43.0	38	32.8	39	33.6
	Not very serious	20	17.2	34	29.3	10	8.6
	Not at all serious	2	1.7	7	6	1	0.9
	Don't know	3	2.6	5	4.3	4	3.4
	I have not heard of this illness	0	0	0	0	0	0
Influenza (*N *= 116)	Very serious	53	45.7	35	30.2	71	61.2
	Fairly serious	48	41.4	49	42.2	36	31
	Not very serious	14	12.1	25	21.6	5	4.3
	Not at all serious	1	0.9	4	3.4	1	0.9
	Don't know	0	0	3	2.6	3	2.6
	I have not heard of this illness	0	0	0	0	0	0
Pertussis (*N *= 116)	Very serious	48	42.5	64	55.2	104	89.7
	Fairly serious	43	37.1	27	23.3	11	9.5
	Not very serious	22	19	18	15.5	1	0.9
	Not at all serious	1	0.9	2	1.7	0	0
	Don't know	1	0.9	5	4.3	0	0
	I have not heard of this illness	0	0	0	0	0	0
Measles (*N *= 116)	Very serious	77	67.2	76	65.5	92	80
	Fairly serious	30	25.9	32	27.6	20	17.4
	Not very serious	4	3.4	2	1.7	2	1.7
	Not at all serious	0	0	0	0	0	0
	Don't know	4	3.4	6	5.2	1	0.9
	I have not heard of this illness	0	0	0	0	0	0
Mumps (*N *= 115 pregnant women, *N *= 116 foetus/baby)	Very serious	65	56.5	68	58.6	90	77.6
	Fairly serious	39	33.9	31	26.7	18	15.5
	Not very serious	4	3.5	6	5.2	4	3.4
	Not at all serious	1	0.9	1	0.9	0	0
	Don't know	6	5.2	10	8.6	4	3.4
	I have not heard of this illness	0	0	0	0	0	0
RSV (*N *= 116)	Very serious	54	46.6	57	49.1	102	87.9
	Fairly serious	40	34.5	31	26.7	10	8.6
	Not very serious	12	10.3	13	11.2	1	0.9
	Not at all serious	2	1.7	3	2.6	0	0
	Don't know	7	6	11	9.5	2	1.7
	I have not heard of this illness	1	0.9	1	0.9	1	0.9
Rubella (*N *= 116)	Very serious	72	62.1	83	71.6	91	78.4
	Fairly serious	34	29.2	24	20.7	19	16.4
	Not very serious	3	2.6	0	0	3	2.6
	Not at all serious	0	0	0	0	0	0
	Don't know	7	6	9	7.8	3	2.6
	I have not heard of this illness	0	0	0	0	0	0

Abbreviation: RSV, respiratory syncytial virus.

Most midwives (*n* = 92, 85.2%) believed a pregnant woman's cultural beliefs influenced their decision making. Midwives were asked to provide further information for their answers. Free‐text responses identified several themes. These included family influence (*n* = 11), misinformation (*n* = 7), COVID‐19 vaccine beliefs (*n* = 4), social media (*n* = 4), ethnic minorities and religious beliefs (*n* = 8), previous negative experiences (*n* = 2) and vaccine content concerns (*n* = 3). Misinformation and social media were frequently linked, with one participant stating, *‘difficult decisions are made even more difficult by misinformation and scaremongering on social media’*. Most midwives (*n* = 98, 89.9%) also believed that a woman's partner and family influenced her decision making. One participant said, *‘partners sometimes discourage vaccination, which can lead to safeguarding concerns due to coercion’*. Most midwives (*n* = 90, 89.1%) felt that their own personal beliefs did not affect the pregnant women's decision making. Themes emerging from qualitative responses midwives said that they focussed on using evidence‐based research (*n* = 8), emphasising informed choice (*n* = 7), remaining impartial (*n* = 8), being respectful of women's decisions (*n* = 5) and professionalism and working within their code of conduct (*n* = 3). Open‐text responses indicated that midwives often had to balance providing information and respecting someone's wishes. For example, one participant said, *‘I strongly agree with vaccines in pregnancy however, I agree more with body autonomy*’.

Midwives were asked about the prevalence of vaccine hesitancy among pregnant women and their colleagues. Most believed vaccine hesitancy was common among pregnant women (*n* = 77, 71.3%) and 38% (*n* = 41) thought it was common among their midwifery colleagues. In free‐text responses concerns were raised about staff hesitancy, with one participant noting ‘*some midwives I work with don't believe in [vaccines] and some attend rallies to incite others not to have them, I feel this is inappropriate for their role’*. Another added ‘*we have some midwives who are anti‐vax. It is difficult to get them to believe the literature regardless of the research. I think we need to have much better training on vaccines and by an expert not a midwife’*. Hesitancy surrounding the COVID‐19 vaccine was also often linked to misinformation. One participant said, *‘I find the COVID vaccine is the one most people are less likely to have due to the short period it was researched and the side effects which are not fully known’*. One participant suggested *‘to deal with misinformation is a significant task’*.

## 4 Discussion

The study explored midwives’ views in relation to supporting the delivery of pregnancy vaccines in Wales within the COM‐B framework. In terms of midwives’ capability, the study identified a gap in vaccination training amongst midwives in Wales, with less than half of midwives receiving training. There was a desire for further education, and midwives identified a clear need for accessible and diverse education delivery methods, such as a pregnancy vaccination toolkit which provides all vaccine information in one place. Practical, adaptable and easily available resources are essential [32]. The study also highlighted that midwives often lack information in diverse languages and that even with translators some women did not understand the information. Language barriers and poor communication are frequently cited as problematic for vaccine acceptance amongst ethnic minorities [32, 42, 43]. Improved provision of high‐quality interpreters, communication aids, and translated materials is therefore needed. Leveraging the influence of community leaders could also help to champion vaccination uptake [43].

Operationally, over two thirds of midwives identified at least one barrier to their opportunity to promoting vaccines, including insufficient time, aligning with Wilson et al.’s [34] London study, which reported that chaotic work environments and time constraints affected midwives’ ability to discuss vaccines effectively. In Wales, workforce shortages within the midwifery sector have been highlighted as a significant challenge affecting various aspects of service provision, including education and training [44]. Overburdened staff may be likely to focus on urgent challenges and become less focussed on preventative public health activities like promoting vaccinations. High workloads may also reduce midwives’ ability to engage and build trusting relationships with women required for effective vaccine conversations [45].

Midwives’ motivation to promote vaccines was also explored. COVID‐19 was viewed as the least serious disease across all population groups. Midwives in England in a very recent qualitative study (published at the time of writing in January 2025) described the end to COVID‐19 restriction, decreased media coverage and decreased visibility of the disease resulting in what they saw as lower perceived seriousness of the disease among pregnant women [46]. Indeed, soon after data collection, the JCVI withdrew the COVID‐19 vaccine from its recommendations for routine offering [6]. In this study midwives identified that for newborns, pertussis was viewed as most serious, which may have been influenced by recent government and media coverage of a pertussis surge in the Wales prior to the survey in 2023/24 [18, 47, 48]. This is not surprising, as mass media exposure of disease outbreaks and vaccines have been associated with increased uptake or changing perceptions of vaccines [49, 50, 51].

Our study revealed a high degree of perceived vaccine hesitancy in Wales, with 71.3% (*n* = 77) of midwives saying they thought vaccine hesitancy was common amongst pregnant women and 38% (*n* = 41) amongst midwives. Strategies to address vaccine hesitancy are urgently needed to promote and achieve higher uptake; however, vaccine hesitancy is complex phenomenon. Cooper and Wiysonge [52] point out that many vaccine hesitant individuals are often not lacking knowledge but may in fact have a high degree of knowledge about vaccines, their components and their side effects. Some vaccine hesitant healthcare workers could fit this profile. Therefore, more education or strategies to improve health literacy may not be an effective intervention for this group. For others, improving knowledge through effective training has been shown to build confidence and empower midwives to challenge vaccine hesitancy [53, 54].

Vaccine hesitancy might be better viewed as a socio‐political phenomenon integrating a person's values, their trust or distrust of institutions and sense of marginalisation [52]. Complicating the issue further is the politicisation of public health policy since COVID‐19 and the volume and ubiquity of online misinformation, which is highly associated with anti‐vax and alternative health views [55, 56, 57]. Vaccine hesitancy is therefore not homogenous across populations or individuals, and it may be context specific. For example, a pregnant woman who might not have always been vaccine hesitant but would not accept a vaccine during pregnancy may be seen as being on a continuum of hesitancy [58]. More research, especially qualitative research, is therefore required to understand the issue in‐depth to assist the development of interventions to reduce vaccine hesitancy among different groups, populations and individuals depending on their values and context. The influence of partners and families was also viewed as important in our study; therefore, midwives should seek to engage them in vaccine discussions to reinforce messages and support informed decision making.

### 4.1 Strengths and Limitations

A key strength of our study was the application of the COM‐B theoretical framework, which provided a structured approach, shaping our thinking and study design in accordance with a recognised model of health behaviour change [39]. This framework enabled us to clearly identify areas for the attention of service providers. The mixed methods approach also allowed us to glean some qualitative depth and meaning behind the descriptive statistics.

Using social media to recruit participants was also useful, as it allowed us to potentially reach a geographically broad group of midwives who might not be accessible through traditional recruitment channels. However, this was limited to reaching only those midwives who are active on social media and within the researchers’ networks (though snowball sharing occurred), limiting the representativeness of the sample.

Our relatively small sample size, which was less than half of our target, meant that any inferential statistical tests were unlikely to be generalisable. However, to answer the research questions in this under‐researched area, descriptive statistics alone were highly informative. For ethical reasons, due to the small population of midwives in Wales (especially midwives from BAME backgrounds), we could not collect any demographic data (such as age or ethnicity) which could potentially identify participants. Most midwives in our study provided care in labour and postnatally and therefore were perhaps less likely to perceive vaccine discussions as a key part of their role, possibly impacting their perceived knowledge and confidence.

## 5 Conclusions

Improved training for midwives, including access to resources to aid communication in different languages may improve midwives’ confidence to discuss vaccines and support an ethnically diverse population; however, operational workload is likely to be an ongoing barrier in the context of overburdened healthcare systems. Vaccine hesitancy is a complex and well documented phenomenon which cannot be oversimplified, and media coverage of disease outbreaks may influence perceived seriousness and uptake. In‐depth qualitative research with midwives and pregnant women is needed to help in the development of interventions to improve vaccine uptake. Future research may also wish to focus on community midwives for whom promoting vaccines is a larger part of their role.

## Author Contributions

**Sara Jones**: conceptualization, writing – original draft, methodology writing – review and editing, supervision, resources. **Kate Lloyd**: conceptualization, writing – original draft, investigation, methodology, writing – review and editing, formal analysis, project administration.

## Ethics Statement

Ethical approval for this research was granted by the Swansea University's School of Health and Social Care Research Ethics Committee.

## Consent

No patients were involved in this research. Informed consent was gathered from midwives who participated in the research.

## Conflicts of Interest

Kate Lloyd was an employee of Public Health Wales as a Specialist Immunisation Nurse during the time the research was conducted.

## Permission to Reproduce Material From Other Sources

Figure 1 was extracted from the World Health Organization (l) tailoring immunisation programmes. Retrieved 18 January 2025 from https://iris.who.int/bitstream/handle/10665/329448/9789289054492‐eng.pdf?sequence=1. This work is available under the Creative Commons Attribution NonCommercial‐ShareAlike 3.0 IGO licence (CC BY‐NC‐SA 3.0 IGO; https://creativecommons.org/licenses/by‐ncsa/3.0/igo). Under the terms of this licence, material may be copied, redistributed and adapted for non‐commercial purposes, provided the work is appropriately cited, as indicated below. In any use of this work, there should be no suggestion that WHO endorses any specific organization, products or services.

## Data Availability

The data that support the findings of this study are available from the corresponding author upon reasonable request.
